# Enhanced histone H3 acetylation of the PD-L1 promoter via the COP1/c-Jun/HDAC3 axis is required for PD-L1 expression in drug-resistant cancer cells

**DOI:** 10.1186/s13046-020-1536-x

**Published:** 2020-02-05

**Authors:** Haifang Wang, Chen Fu, Jun Du, Hongsheng Wang, Rui He, Xiaofeng Yin, Haixia Li, Xin Li, Hongxia Wang, Kui Li, Lei Zheng, Zongcai Liu, Yurong Qiu

**Affiliations:** 1 Laboratory Medicine Center, Nanfang Hospital, Southern Medical University, Guangzhou, 510515 China; 2 Department of Microbial and Biochemical Pharmacy, School of Pharmaceutical Sciences, Sun Yat-sen University, Guangzhou, 510006 China; 3 The Laboratory of Endocrinology and Metabolism, Guangzhou Women and Children’s Medical Center, Guangzhou Medical University, Guangzhou, 510623 China; 4 Organ Transplantation Center, The First Affiliated Hospital, Sun Yat-sen University, Guangzhou, 510080 China; 5 Guangzhou Huayin Medical Laboratory Center Co., Ltd., Guangzhou, 510515 China

**Keywords:** PD-L1, Drug resistance, c-Jun, Histone acetylation, HDAC3

## Abstract

**Background:**

Drug resistance is a major obstacle to treating cancers because it desensitizes cancer cells to chemotherapy. Recently, attention has been focused on changes in the tumor immune landscape after the acquisition of drug resistance. Programmed death-ligand-1 (PD-L1) is an immune suppressor that inhibits T cell-based immunity. Evidence has shown that acquired chemoresistance is associated with increased PD-L1 expression in cancer cells. However, the underlying mechanism is still largely unknown.

**Methods:**

PD-L1 expression in three drug-resistant A549/CDDP, MCF7/ADR and HepG2/ADR cell lines was detected by qRT-PCR, western blotting and flow cytometry, and a T cell proliferation assay was performed to test its functional significance. Then, the potential roles of JNK/c-Jun, histone H3 acetylation, histone deacetylase 3 (HDAC3) and the E3 ligase COP1 in the PD-L1 increase were explored through ChIP assays and gain- and loss-of-function gene studies. Furthermore, murine xenograft tumor models were used to verify the role of JNK/c-Jun and HDAC3 in PD-L1 expression in A549/CDDP cells in vivo. Finally, the correlations of PD-L1, c-Jun and HDAC3 expression in clinical cisplatin-sensitive and cisplatin-resistant non-small cell lung cancer (NSCLC) tissues were analyzed by immunohistochemistry and Pearson’s correlation coefficient.

**Results:**

PD-L1 expression was significantly increased in A549/CDDP, MCF7/ADR and HepG2/ADR cells and was attributed mainly to enhanced JNK/c-Jun signaling activation. Mechanistically, decreased COP1 increased c-Jun accumulation, which subsequently inhibited HDAC3 expression and thereby enhanced histone H3 acetylation of the PD-L1 promoter. Furthermore, PD-L1 expression could be inhibited by JNK/c-Jun inhibition or HDAC3 overexpression in vivo, which could largely reverse inhibited CD3^+^ T cell proliferation in vitro. PD-L1 expression was significantly increased in the cisplatin-resistant clinical NSCLC samples and positively correlated with c-Jun expression but negatively correlated with HDAC3 expression.

**Conclusions:**

Enhanced histone H3 acetylation of the PD-L1 promoter via the COP1/c-Jun/HDAC3 axis was crucial for the PD-L1 increase in drug-resistant cancer cells. Our study reveals a novel regulatory network for the PD-L1 increase in drug-resistant cancer cells and that combined PD-L1-targeting strategies could improve T cell-based immunity in drug-resistant cancers.

## Introduction

Cancer is currently the second leading cause of death globally, with an estimated 18.1 million new cases and 9.6 million deaths in 2018 worldwide [1]. Chemotherapy is one of the most adopted strategies to treat cancers. However, despite a positive initial response, most patients eventually suffer from recurrence due to drug resistance [2]. Previously, drug resistance was mainly known as a mechanism to prevent cancer cells from being effectively eliminated by chemotherapeutic drugs. However, extensive attention has recently been focused on changes in the tumor immune landscape after the acquisition of drug resistance, and the related findings can help to improve the treatment of drug-resistant cancers from the aspect of tumor immunity [3, 4].

Programmed death-ligand-1 (PD-L1) is one of the most important immune checkpoint molecules and is widely expressed on the surface of tumor cells [5]. PD-L1 significantly inhibits the proliferation and function of T cells through binding with programmed cell-death protein 1 (PD-1) on T cells; thus, its aberrant expression is closely associated with impaired tumor immunity and poor prognosis in patients [5]. Recently, PD-L1/PD-1 axis blockade has been suggested as a potent strategy against multiple malignancies, including non-small cell lung cancer (NSCLC), hepatocellular carcinoma (HCC) and breast cancer (BC) [6–9], and this highlights the importance of PD-L1 in promoting tumor progression through immunosuppression.

Recently, accumulating evidence has shown that acquired resistance to chemotherapeutic drugs such as platinum, epidermal growth factor receptor tyramine kinase (EGFR-TK) inhibitors, and anaplastic lymphoma kinase (ALK) inhibitors is associated with increased PD-L1 expression in cancer cells [10–12]. Acquired drug resistance to ALK inhibitors or sorafenib induces PD-L1 expression in cancer cells [12, 13], which suggests the causality between drug resistance and increased PD-L1. In addition, other studies demonstrated that increased PD-L1 expression can mediate or maintain the drug resistance of cancer cells [14–16]. These findings have revealed the complexity of the relationship between acquired drug resistance and increased PD-L1 in cancer cells. However, the underlying mechanism of increased PD-L1 in drug-resistant cancer cells remains largely unknown.

In this work, we investigated PD-L1 expression and the underlying mechanisms in cisplatin (CDDP)-resistant human non-small cell lung cancer cells (A549/CDDP), doxorubicin (ADR)-resistant human breast cancer cells (MCF7/ADR) and human hepatocellular carcinoma cells (HepG2/ADR). We demonstrated that PD-L1 expression was significantly increased in the above drug-resistant cancer cells, and this change was attributed mainly to enhanced JNK/c-Jun signaling activation. Mechanistically, a decrease in the E3 ligase constitutive photomorphogenesis protein 1 (COP1) increased c-Jun accumulation, which subsequently inhibited histone deacetylase 3 (HDAC3) expression and thus enhanced histone H3 acetylation of the PD-L1 promoter. Furthermore, PD-L1 expression could be inhibited by JNK/c-Jun inhibition or HDAC3 overexpression in vivo, which could largely reverse the inhibited CD3^+^ T cell proliferation in vitro. In clinical NSCLC samples, PD-L1 expression was significantly increased in the cisplatin-resistant group, and PD-L1 expression was positively correlated with c-Jun expression but negatively correlated with HDAC3 expression. In conclusion, enhanced histone H3 acetylation of the PD-L1 promoter via the COP1/c-Jun/HDAC3 axis was crucial for the PD-L1 increase in drug-resistant cancer cells. Our study reveals a novel regulatory network for the PD-L1 increase in drug-resistant cancer cells and that combined PD-L1-targeting strategies could improve T cell-based immunity in drug-resistant cancers.

## Materials and methods

### Chemicals and reagents

Primary antibodies against PD-L1 (#13684), p-JNK (#9251), JNK (#9252), p-c-Jun (#9261), c-Jun (#9165), HDAC3 (#3949), ubiquitin (#3933) were obtained from Cell Signaling Technology (USA). PE conjugated primary antibody PD-L1 (#393608) and CD3 (#300308) were purchased from Biolegend (USA). Purified human PD-L1 antibody (#329747) used for PD-L1 blocking was obtained from BioLegend (USA). CD3^+^ T cells positive selection kits (#130–050-101) were purchased from Miltenyi Biotech (Germany), Purified anti-CD3 (#566685, Clone: OKT3) and anti-CD28 antibodies (#555728, Clone: CD28.2) were purchased from BD Biosciences (USA). Carboxyfluorescein succinimidyl ester (CFSE, #C34554), DAPI (#D21490) and lipofectamine 3000 (#L3000015) were purchased from Invitrogen (USA). JNK inhibitor SP600125 (#S1460) and proteasome inhibitor MG132 (#S2619) were purchased from Selleck Chemicals (USA); JNKs agonist anisomycin (#HY-18982) was purchased from Medchem Express (USA). PrimeScript® RT reagent Kit (#DRR037A) and SYBR® Premix Ex TaqTM (#DRR420A) were products of TaKaRa. E.Z.N.A® HP (Japan). Total RNA Kit (#R1034) was purchased from Omega Bio-Tek (USA). Small interfering RNA (siRNA) against human c-Jun, c-Fos, S6K (ribosomal protein S6 kinase), Stat1 (signal transducers and activators of transcription 1), Stat3 (signal transducers and activators of transcription 3), IRF1 (interferon regulatory factor 1) and negative control were purchased from RiboBio (China). The plasmid vector (pEnter), Flag- and His- tagged c-Jun overexpression plasmid (pEnter-c-Jun, #CH836318) and COP1 overexpression plasmid (pEnter-COP1, #CH884210) were purchased from Vigene Biosciences (China). The lentiviral null vector (pReceiver-Lv233) and HDAC3 overexpression vector (pReceiver-Lv233-HDAC3) were purchased from GeneCopoeia Inc. (USA).

### Cell lines and cell culture

Parental cancer cells (A549, MCF-7, HepG2) and their drug-resistant counterparts (A549/CDDP, MCF-7/ADR, and HepG2/ADR) were kindly provided by the Cancer Institute & Hospital, Chinese Academy of Medical Sciences (Beijing, China). All cells were cultured in RPMI-1640 or DMEM supplemented with 10% FBS, 100 U/mL penicillin, and 100 g/mL streptomycin (Invitrogen, USA) in a humidified atmosphere of 5% CO2 at 37 °C.

### Patients and tumor tissues

To examine PD-L1 protein expression in NSCLC tissues sensitive and resistant to cisplatin treatment, we retrospectively obtained 90 cases of NSCLC tissues from the First Affiliated Hospital of Sun Yat-sen University during 2014 ~ 2017. NSCLC tissues were paraffin embedded and sectioned for immunohistochemistry, and all tumor tissues were pathologically diagnosed as NSCLC according to the WHO classification criteria. All patients received 1 to 3 courses of cisplatin treatment before surgery. The clinical characteristics of the patients are shown in Additional file 1: Table S1. According to the Response Evaluation Criteria In Solid Tumors (RECIST), 45 tumor samples from patients with a 30% or more decrease in the entire tumor burden after cisplatin therapy were considered to be sensitive to cisplatin, while another 45 tumor samples from patients with a 20% increase in the entire tumor burden or appearance of new lesions after cisplatin therapy were considered to be resistant to cisplatin [10]. This study was approved by the Ethics Committee of the First Affiliated Hospital of Sun Yat-sen University, and all methods were carried out in accordance with the approved guidelines. Written informed consent was signed and documented by all the patients who participated in the study.

### Cell transfection

For transfection, cells were seeded on a 6-well plate (2 × 10^5^ cells/well) and cultured for 12 h. Then cells were transfected with 2 μg plasmid or 100 pmol siRNA mixed with lipofectamine 3000 reagent in the complete medium with 10% FBS according to the manufacturer’s instructions, and then incubated for indicated time before harvest.

### Quantitative real-time PCR

Quantitative real-time PCR was performed as previously described [17]. The primer sequences used in each reaction were listed as Additional file 1: Table S2.

### Western blot analysis

Western blotting was performed as previously described [18]. Notably, c-Jun and COP1 were detected in the western blotting assays using anti-c-Jun and anti-COP1 antibodies, respectively, but not anti-His or anti-Flag antibodies when the overexpression plasmids were used.

For nuclear and cytoplasmic protein detection, nuclear and cytoplasmic proteins were extracted using a nuclear/cytosol fractionation kit (#P0028, Beyotime, China) according to the manufacturer’s instructions, and the samples were then examined by western blotting.

To detect c-Jun ubiquitination, cells were treated with or without MG132 (10 μM) for 8 h. Subsequently, the cells were lysed and immunoprecipitated with a primary antibody against c-Jun or rabbit control IgG, and equal amounts of immunoprecipitates were then subjected to immunoblot analysis using an anti-ubiquitin mAb to detect ubiquitin.

### Flow cytometry

To detect antigens on the cell membrane by flow cytometry, cell suspensions were washed with PBS and then directly incubated with indicated antibodies (such as anti-PD-L1 antibodies) or isotype controls for 1 h at 4 °C. Subsequently, the cells were washed and resuspended with PBS, and then fluorescence data were collected on a flow cytometry machine (Millipore, USA). The data were analyzed using FlowJo 7.6.1 software.

### Immunofluorescence microscopy

To detect the localization and expression of p-c-Jun in cancer cells, cells were seeded on 96-well plate (3 × 10^3^ cells/well) overnight, and then were fixed with 4% paraformaldehyde for 20 min and permeated by 1% Triton-X100 for 15 min. Subsequently, cells were blocked with 10% normal goat serum for 30 min at 37 °C and incubated with antibodies against p-c-Jun (1:100 dilution) at 4 °C overnight. After washing with PBS, slides were incubated with FITC conjugated secondary antibodies (1:1000 dilution) and counter stained with DAPI (10 mg/ml). The expression of p-c-Jun was detected by High Throughput Screening equipment (ArrayScan VTI 600 plus, Thermo).

### Chromatin immunoprecipitation

Chromatin immunoprecipitation assays were performed according to the manufacturer’s instructions of the Acetyl-Histone H3 Immunoprecipitation (ChIP) Assay Kit (#17–245, Millipore, USA). Briefly, cells were crosslinked by 1% formaldehyde incubation and then sonicated on ice to shear the DNA to lengths between 200 and 1000 base pairs. Soluble chromatin fragments of 200 to 1000 bp in length were incubated with 5 μg of anti-acetyl-histone H3 antibodies at 4 °C overnight. Normal rabbit IgG was used as a negative control for validating the ChIP assay. Isolated DNA fragments were purified, and quantitative PCR was performed using 2 μl of DNA in triplicate. ChIP primers covering 1800 bp upstream of the human PD-L1 gene start codon were designed by NCBI-Blast software. Amplicons were between 60 and 150 base pairs, and the primers were as follows: primer 1 (− 1178 bp to − 1117 bp), forward 5′- GCT GGG CCC AAA CCC TAT T − 3′ and reverse 5′-TTT GGC AGG AGC ATG GAG TT-3′; primer 2 (− 455 bp to − 356 bp), forward 5′-ATG GGT CTG CTG CTG ACT TT-3′ and reverse 5′-GGC GTC CCC CTT TCT GAT AA-3′; primer 3 (− 105 bp to − 32 bp), forward 5′-ACT GAA AGC TTC CGC CGA TT-3′ and reverse 5′-CCC AAG GCA GCA AAT CCA GT-3′. ChIP-qPCR result was calculated using the ΔΔct method. Briefly, each ChIP fractions’ Ct value was normalized to the input DNA fraction Ct value to account for chromatin sample preparation differences (Δct _normalized ChIP_). Fold changes in H3 acetylation in the PD-L1 promoter of drug-resistant cancer cells were calculated by 2^−ΔΔCt^, where ΔΔCt = ΔCt _[drug-resistant cancer cells: normalized ChIP]_ − ΔCt _[drug-sensitive cancer cells: normalized ChIP]_.

### Immunohistochemical examination

Immunohistochemical examination was performed as previously described [ 17]. Immunohistochemical sections were observed and images were captured for 5 random fields by two pathologists without knowing the patients’ clinical information under a light microscope (Nikon, Japan) at a magnification × 20. The staining intensity was assessed using a modified quickscore method on a scale of 0–3 as negative (0), weak (1), medium (2) or strong (3). The extent of the staining, defined as the percentage of positive stained areas of cancer cells per the whole tumor area, was scored on a scale of 0 (0%), 1 (1–25%), 2 (26–50%), 3 (51–75%) and 4 (76–100%). An overall protein expression score (overall score range, 0–12) was calculated by multiplying the intensity and positivity scores according to our previous study [19].

### Establishment of A549/CDDP cells with stable HDAC3 overexpression

A549/CDDP cells were transfected with lentiviruses with HDAC3 expression vectors (pReceiver-HDAC3) or their control null vectors (pReceiver) at a multiplicity of infection of 100 transfecting units per cell in the presence of 5 mg/ml polybrene. The transfected A549/CDDP cells were selected with puromycin (1 μg/ml) for 10~14 days. The surviving cells were then picked out and reseeded into a 96-well plate for cell clone formation and expansion. The expanded monoclonal cell populations (named A549/CDDP^HDAC3^ and A549/CDDP^pReceiver^) were collected and stored for further study.

### Animal studies

Six-week-old female BALB/C nude mice were obtained from the Animal Experimental Center of Southern Medical University (Guangzhou, China). The procedures for the handling and care of the mice were approved by the Animal Experimentation Ethics Committee of Southern Medical University. In total, 1 × 10^7^ A549, A549/CDDP, A549/CDDP^pReceiver^ or A549/CDDP^HDAC3^ cells in Matrigel (BD Biosciences, USA) were injected into the right flanks of nude mice to form xenograft tumors. When the tumor volumes reached ~ 100 mm^3^, mice bearing A549/CDDP tumors were treated with SP600125 (15 mg/kg) in PPCES vehicle (30% PEG-400, 20% polypropylene glycol, 15% Cremophor EL, 5% ethanol and 30% saline) or PPCES vehicle alone every 4 days by intragastrical gavage for 2 weeks. At the end, the tumors were collected and then digested to prepare a single cell suspension for cell surface PD-L1 detection and CD3^+^ T cell proliferation assays.

### CD3^+^ T cell proliferation assay

A CD3^+^ T cell proliferation assay was performed as previously described [20]. Briefly, CD3^+^ T cells were isolated from healthy donor PBMCs using positive selection kits and labeled with CFSE. Then, 3 × 10^5^ CFSE-labeled CD3^+^ T cells were cocultured with 1 × 10^3^ cultured or tumor-derived cancer cells in 96-well plates. Next, the cocultured T cells were stimulated by the addition of anti-CD3 (3 μg/ml) and anti-CD28 antibodies (3 μg/ml). After 3 days, the cells were harvested and stained with a PE-conjugated anti-CD3 antibody, and T cell proliferation was determined by measuring the CFSE dilution using flow cytometry after gating on the CD3^+^ cell populations.

### Statistical analysis

Results were expressed as mean ± standard deviation (SD) of three independent experiments unless otherwise specified. Student’s t test and one-way ANOVA were performed to compare the differences between groups. The correlations of PD-L1, c-Jun and HDAC3 expression in tumor tissues were analyzed by Pearson’s correlation coefficient. Statistical analyses were performed using GraphPad Prism Software Version 5.0 (GraphPad Software Inc., CA, USA). All experiments were performed independently in triplicate. *P* < 0.05 was considered as statistically significant. ^*^P ≤ 0.05, ^**^P ≤ 0.01, ^***^P ≤ 0.001.

## Results

### PD-L1 expression is increased in drug-resistant A549/CDDP, MCF7/ADR and HepG2/ADR cells and suppresses CD3^+^ T cell proliferation in vitro

First, we confirmed that A549/CDDP cells were resistant to cisplatin and that MCF7/ADR and HepG2/ADR cells were resistant to doxorubicin compared with their parental cells, and these drug-resistant cancer cells had significantly higher IC_50_ values (Additional file 1: Figure S1).

**Fig. 1:**
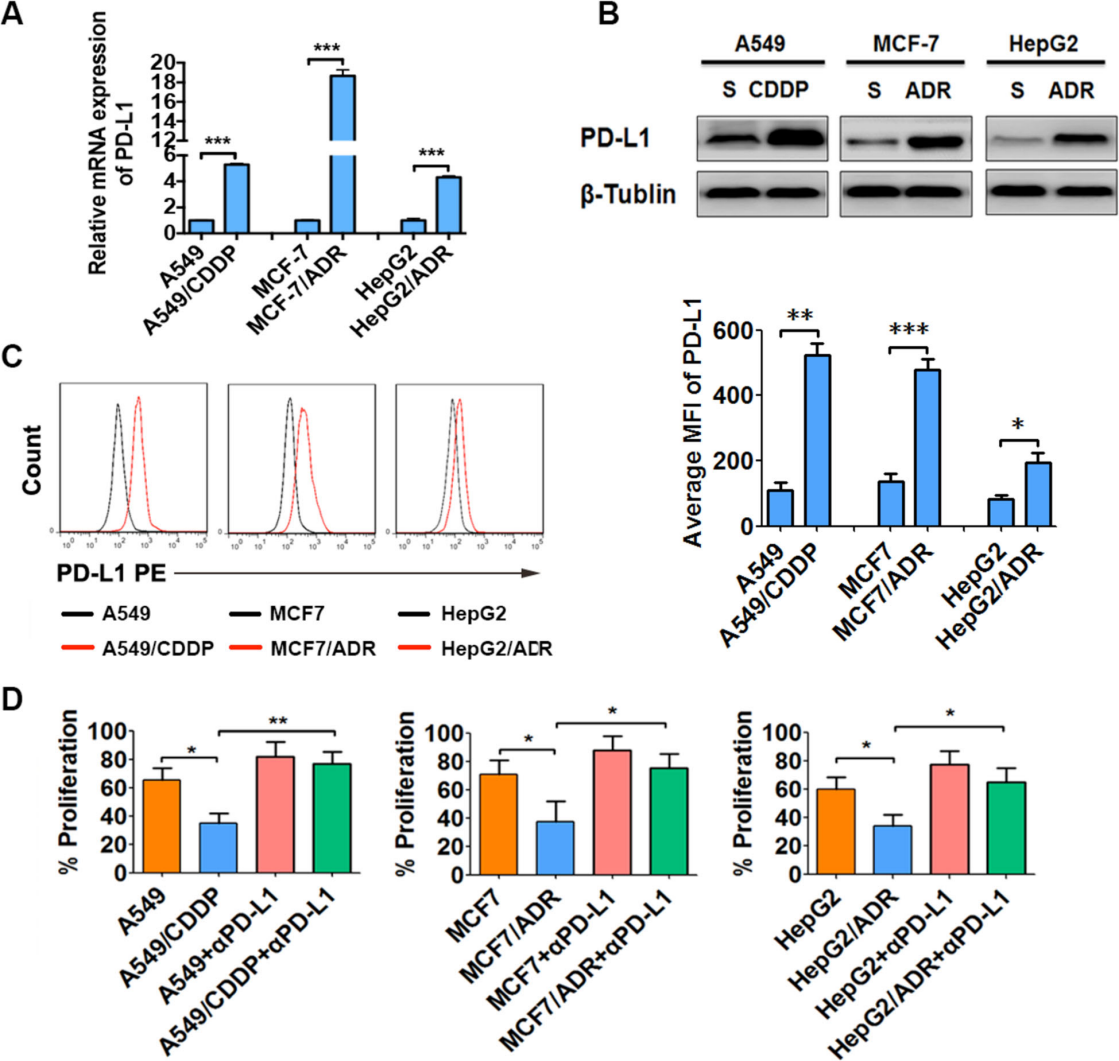
PD-L1 is functionally increased in drug-resistant A549/CDDP, MCF7/ADR and HepG2/ADR cells. PD-L1 expression in drug**-**resistant A549/CDDP, MCF7/ADR and HepG2/ADR cells and their parental cancer cells were detected by qRT-PCR (**a**), western blotting (**b**) and flow cytometry analysis (**c**, left). The average mean fluorescence intensity (MFI) of PD-L1 expression in these cells detected by flow cytometry was calculated and compared (**c**, right). All of the above experiments were performed independently in triplicate (S: drug-sensitive; CDDP: cisplatin-resistant; ADR: doxorubicin-resistant). **d** CD3^+^ T cells isolated from PBMCs were prelabeled with CFSE and cocultured with drug**-**resistant cancer cells or their parental cells with or without anti-PD-L1 antibodies (αPD-L1). After stimulation with anti-CD3/CD28 antibodies for 72 h, cell proliferation was measured using flow cytometry. ^*^P ≤ 0.05, ^**^P ≤ 0.01, ^***^P ≤ 0.001

Next, we detected PD-L1 expression in the drug-resistant cancer cells and their parental counterparts by qRT-PCR and western blotting. PD-L1 expression was significantly increased in drug-resistant cancer cells compared with their parental counterparts (Fig. 1a and b), and these findings were confirmed by flow cytometry (Fig. 1c). These results demonstrated that PD-L1 expression was increased in drug-resistant A549/CDDP, MCF7/ADR, and HepG2/ADR cells. To determine if the PD-L1 increase was functionally important, we detected the effect of increased PD-L1 on CD3^+^ T cell proliferation. It was demonstrated that these drug-resistant cancer cells inhibited significantly more proliferation in CD3^+^ T cells than their parental cancer cells (Fig. 1d). In addition, after PD-L1 blockade by specific antibodies, the increased CD3^+^ T cell proliferation inhibition by drug-resistant cancer cells was largely reversed, while CD3^+^ T cell proliferation in the parental cells was only slightly changed after PD-L1 blockade, which might be due to low endogenous PD-L1 expression (Fig. 1d). The above results collectively demonstrated that PD-L1 expression was increased in drug-resistant A549/CDDP, MCF7/ADR and HepG2/ADR cells, which suppressed CD3^+^ T cell proliferation in vitro.

### c-Jun is crucial for the enhanced PD-L1 expression in drug-resistant A549/CDDP, MCF7/ADR and HepG2/ADR cells

Previous studies have shown that transcription factors such as c-Jun, c-Fos, STAT1/3, S6K and IRF are involved in PD-L1 expression in cancer cells [21]. In our study, it was demonstrated that c-Jun knockdown could potently decrease PD-L1 expression in both A549/CDDP and MCF7/ADR cells, suggesting that c-Jun might be a potential shared regulator of PD-L1 expression in these drug-resistant cancer cells (Additional file 1: Figure S2).

**Fig. 2:**
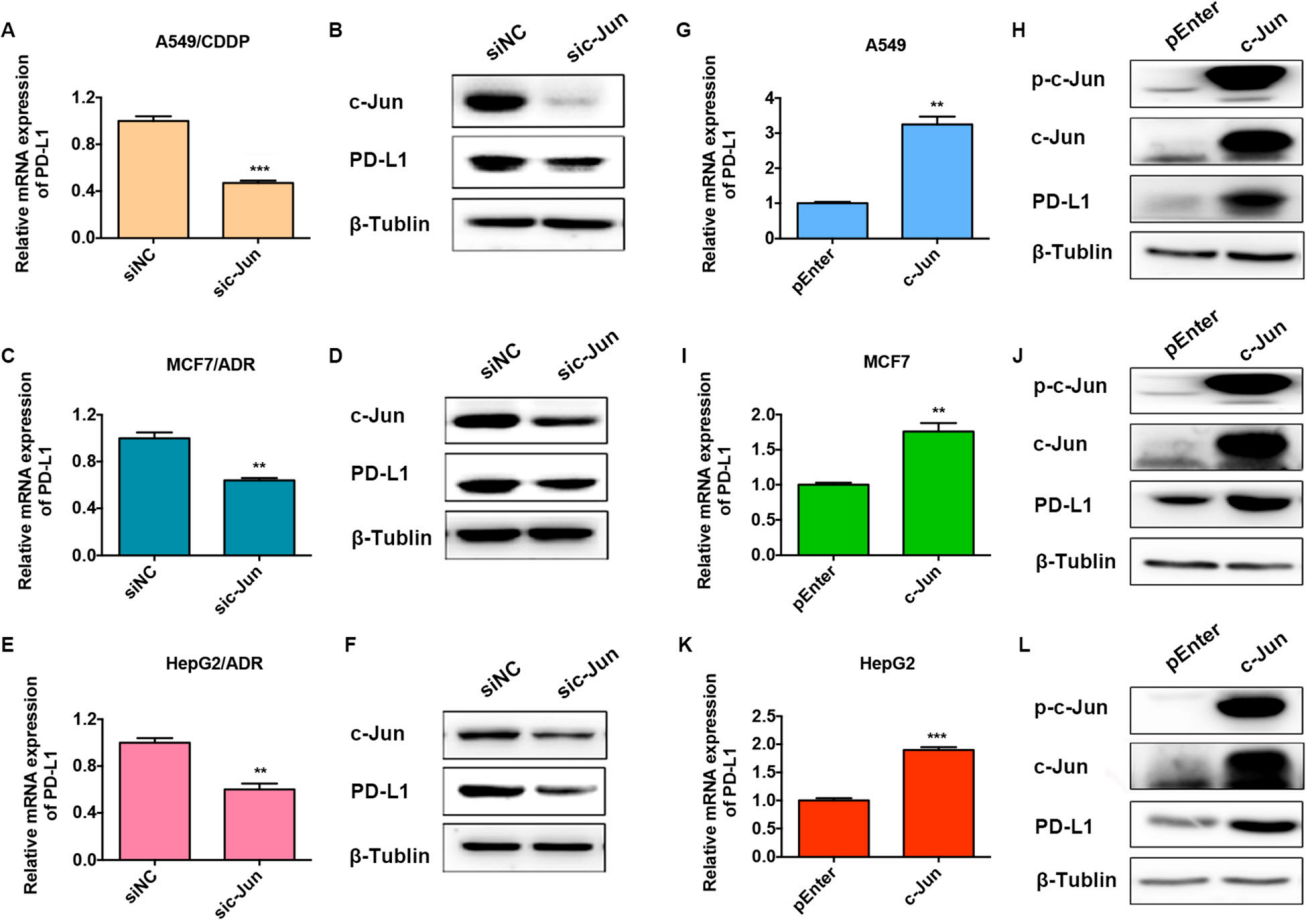
c-Jun is crucial for the enhanced PD-L1 expression in drug-resistant A549/CDDP, MCF7/ADR and HepG2/ADR cells. Drug-resistant A549/CDDP (**a** and **b**), MCF7/ADR (**c** and **d**) and HepG2/ADR (**e** and **f**) cells were transfected with c-Jun-targeting siRNAs for 24 h (for qRT-PCR) or 48 h (for western blotting), and PD-L1 expression was detected by qRT-PCR or western blotting. Parental A549 (**g** and **h**), MCF7 (**i** and **j**) and HepG2 (**k** and **l**) cells were transfected with c-Jun expression plasmids for 24 h (for qRT-PCR) or 48 h (for western blotting), and PD-L1 expression was detected by qRT-PCR or western blotting. All experiments were performed independently in triplicate. ^*^P ≤ 0.05, ^**^P ≤ 0.01, ^***^P ≤ 0.001

We subsequently determined the role of c-Jun in the expression of PD-L1 in cancer cells in our study. The results confirmed that c-Jun knockdown markedly inhibited PD-L1 mRNA and protein expression in all drug-resistant cancer cells (Fig. 2a-f), while c-Jun overexpression significantly increased PD-L1 mRNA and protein expression in all of the parental counterparts (Fig. 2g-l). These findings collectively demonstrated that c-Jun was crucial for the increased PD-L1 expression in drug-resistant A549/CDDP, MCF7/ADR and HepG2/ADR cells.

### JNK/c-Jun signaling activation is enhanced and mediates the PD-L1 increase in drug-resistant A549/CDDP, MCF7/ADR and HepG2/ADR cells

**Fig. 3:**
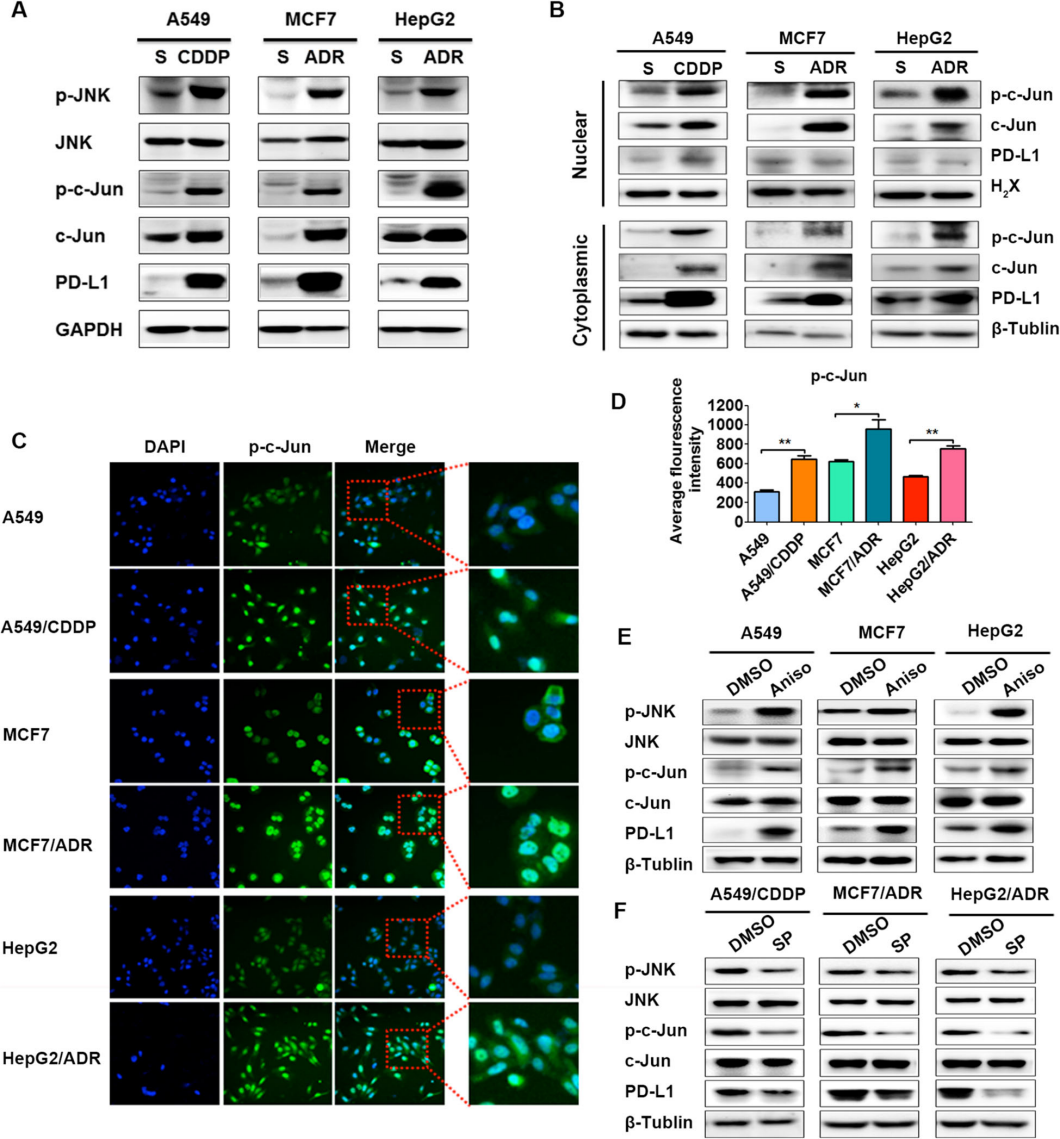
JNK/c-Jun signaling activation is enhanced and mediates the PD-L1 increase in drug-resistant A549/CDDP, MCF7/ADR and HepG2/ADR cells. Drug-resistant cancer cells and their parental cancer cells were used to detect JNK/c-Jun signaling activation. **a** p-JNK, JNK, p-c-Jun, c-Jun and PD-L1 expression was detected by western blotting, and **b** c-Jun and p-c-Jun expression in the nuclear and cytoplasmic fractions of drug-resistant cancer cells and their parental cancer cells was detected by western blotting. Representative immunofluorescence images of p-c-Jun expression and its subcellular location in drug-resistant cancer cells and their parental cancer cells are shown (**c**), and the average fluorescence intensity was measured and compared (**d**). Images were taken at × 20 magnification, and the specified fields were taken at × 40 magnification. Parental cancer cells were treated with the JNK agonist anisomycin (Aniso, 10 μM) for 48 h (**e**), while drug-resistant cancer cells were treated with the JNK inhibitor SP6000125 (SP, 10 μM) for 48 h (**f**). PD-L1 expression was then determined by western blotting. All experiments were performed independently in triplicate. ^*^P ≤ 0.05, ^**^P ≤ 0.01, ^***^P ≤ 0.001

Given the importance of c-Jun and that c-Jun is activated by JNK kinase via phosphorylation [22], we compared JNK/c-Jun signaling activation between drug-resistant cancer cells and their parental cancer cells in our study. It was found that phosphorylated JNK and c-Jun protein levels, as well as total c-Jun but not total JNK protein levels, were markedly higher in drug-resistant cancer cells than in their parental counterparts (Fig. 3a). As a transcription factor, phosphorylated c-Jun begins to translocate into the nucleus after phosphorylation and activation by JNK. Thus, we detected the nuclear and cytoplasmic distribution of phosphorylated c-Jun (p-c-Jun). The results showed that both the nuclear and cytoplasmic levels of p-c-Jun were significantly higher in drug-resistant cancer cells, while p-c-Jun was located mostly in the nuclei of the cancer cells (Fig. 3b). In addition, our immunofluorescence images and analyses further confirmed more nuclear p-c-Jun protein accumulation in the drug-resistant cancer cells (Fig. 3c and d). The above results demonstrated that JNK/c-Jun signaling activation was greater in drug-resistant cells in our study. Next, the JNK agonist anisomycin and the JNK inhibitor SP600125 were used to confirm the role of JNK/c-Jun signaling regulation in PD-L1 expression in cancer cells in our study. It was demonstrated that JNK/c-Jun signaling activation by anisomycin could significantly increase PD-L1 expression in the parental cancer cells, while JNK/c-Jun signaling inhibition by SP600125 could markedly decrease PD-L1 expression in the drug-resistant cancer cells (Fig. 3e and f), which confirmed the crucial role of JNK/c-Jun signaling. The above results collectively demonstrated that JNK/c-Jun signaling activation was enhanced and mediated the PD-L1 increase in drug-resistant A549/CDDP, MCF7/ADR and HepG2/ADR cells.

### Histone H3 acetylation of the PD-L1 promoter is increased and mediated by the c-Jun/HDAC3 axis in drug-resistant A549/CDDP, MCF7/ADR and HepG2/ADR cells

**Fig. 4:**
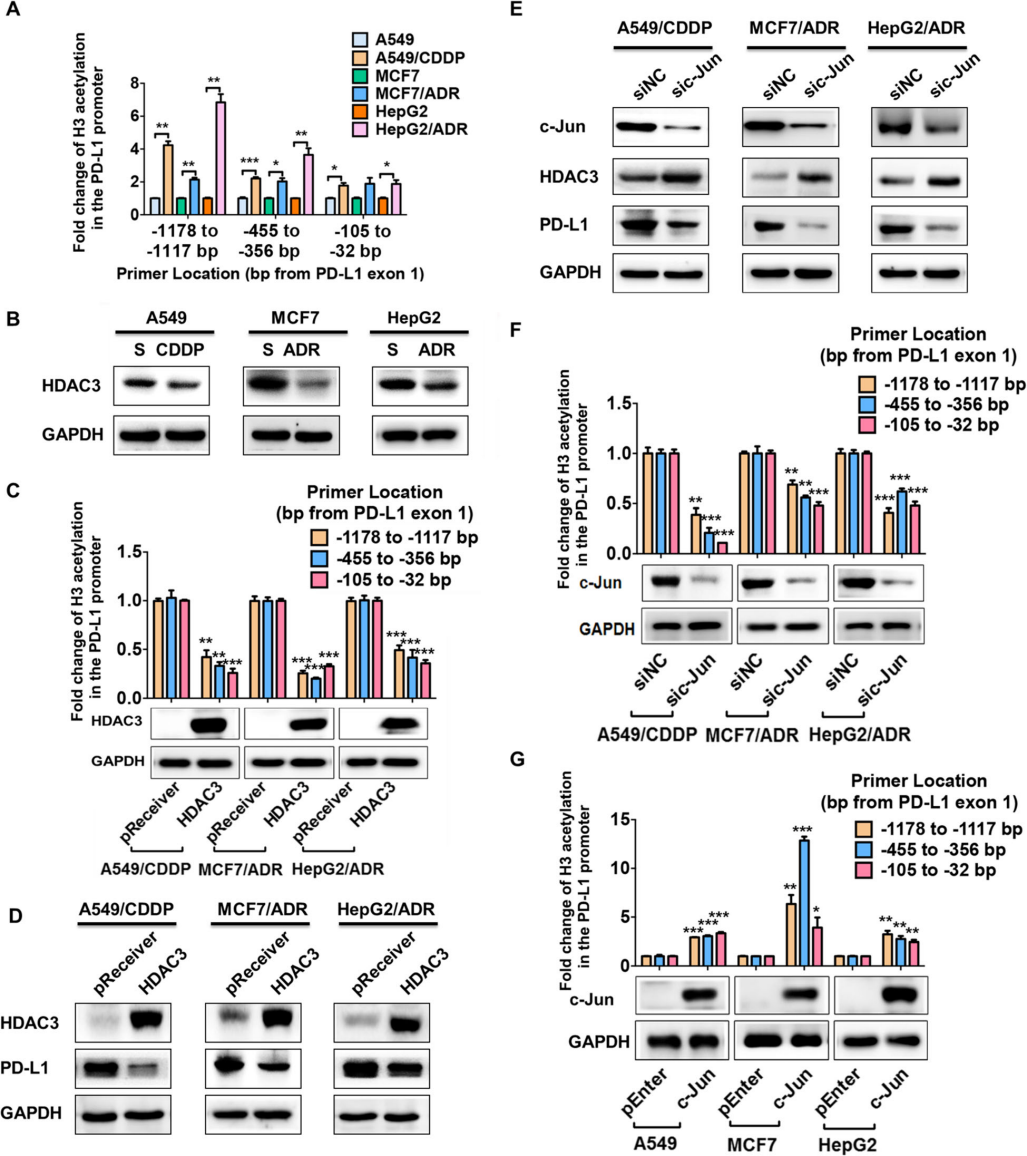
Histone H3 acetylation in the PD-L1 promoter is increased and mediated by the c-Jun/HDAC3 axis. Cells were chromatin immunoprecipitated for acetylated histone H3 or IgG, and the DNA pull-down samples were then quantified by qRT-PCR. **a** The fold enrichment of histone H3 acetylation in the PD-L1 promoter DNA fragments (− 1178 bp to − 1117 bp, − 455 bp to − 356 bp, and − 105 bp to − 32 bp from PD-L1 exon 1) was detected and compared in the drug-resistant and parental cancer cells. **b** Parental cancer cells and drug-resistant cancer cells were collected, and HDAC3 expression was detected by western blotting. A549/CDDP, MCF-7/ADR, and HepG2/ADR cells were transfected with HDAC3 expression vector (HDAC3) or control vector (pReceiver), then the fold enrichment in histone H3 acetylation in the PD-L1 promoter was detected by ChIP assays (**c**, upper), HDAC3 expression was confirmed by western blotting (**c**, lower), and PD-L1 protein expression was detected by western blotting (**d**). **e** Drug-resistant cancer cells were transfected with c-Jun-targeting siRNAs for 48 h, and HDAC3 and PD-L1 expression was detected by western blotting. Drug-resistant cancer cells were transfected with c-Jun-targeting siRNAs or c-Jun expression plasmids for 24 h, then the fold enrichment in histone H3 acetylation in the PD-L1 promoter was detected by ChIP analysis (**f** and **g**, upper), and c-Jun expression was confirmed by western blotting (**f** and **g**, lower). ^*^P ≤ 0.05, ^**^P ≤ 0.01, ^***^P ≤ 0.001

Epigenetic modifications, such as DNA methylation and histone acetylation, are frequently involved in regulating PD-L1 expression in cancer cells [23–25]. First, a total of 18 CpG islands in the PD-L1 promoter region were sequenced, and the DNA methylation of the PD-L1 promoter region was barely changed in the drug-resistant cancer cells in our study (Additional file 1: Figure S3). Next, we compared the histone acetylation of the PD-L1 promoter region between the drug-resistant cancer cells and their parental counterparts by chromatin immunoprecipitation assays. The levels of histone H3 acetylation in the PD-L1 promoter region (− 1178 bp to − 1117 bp, − 455 bp to − 356 bp, and − 105 bp to − 32 bp from PD-L1 exon 1) of these drug-resistant cancer cells were significantly increased (Fig. 4a).

Histone acetylation is tightly regulated by HDACs in cancer cells [26]. In addition, HDACs have been reported to regulate PD-L1 transcription [27]. Thus, HDAC inhibitors were used to determine whether HDACs were involved in the PD-L1 increase in drug-resistant cancer cells. It was shown that only the pan-HDAC inhibitor SAHA and the HDAC3-specific inhibitor RGFP966 markedly increased PD-L1 expression in all the parental cancer cells in our study (Additional file 1: Figure S4), suggesting the important role of HDAC3. Next, HDAC3 expression was compared in the drug-resistant cancer cells and parental cells. The drug-resistant cancer cells expressed lower HDAC3 levels than their parental counterparts (Fig. 4b). Furthermore, HDAC3 overexpression significantly decreased histone H3 acetylation of the PD-L1 promoter region and PD-L1 expression in the drug-resistant cancer cells (Fig. 4c and d).

Given the pivotal role of c-Jun in PD-L1 expression, we next studied whether c-Jun regulated HDAC3 expression. It was demonstrated that c-Jun knockdown by siRNAs increased HDAC3 expression in drug-resistant cancer cells in our study (Fig. 4e). We further detected that c-Jun knockdown markedly decreased the histone H3 acetylation levels in the PD-L1 promoter region of these drug-resistant cells, while c-Jun overexpression significantly increased histone H3 acetylation in the PD-L1 promoter region of the parental counterparts (Fig. 4f and g). The above results collectively demonstrated that histone H3 acetylation of the PD-L1 promoter region was increased and mediated by the c-Jun/HDAC3 axis in drug-resistant A549/CDDP, MCF7/ADR and HepG2/ADR cells.

### Decreased levels of the E3 ligase COP1 promote c-Jun protein accumulation in drug-resistant A549/CDDP, MCF7/ADR and HepG2/ADR cells

**Fig. 5:**
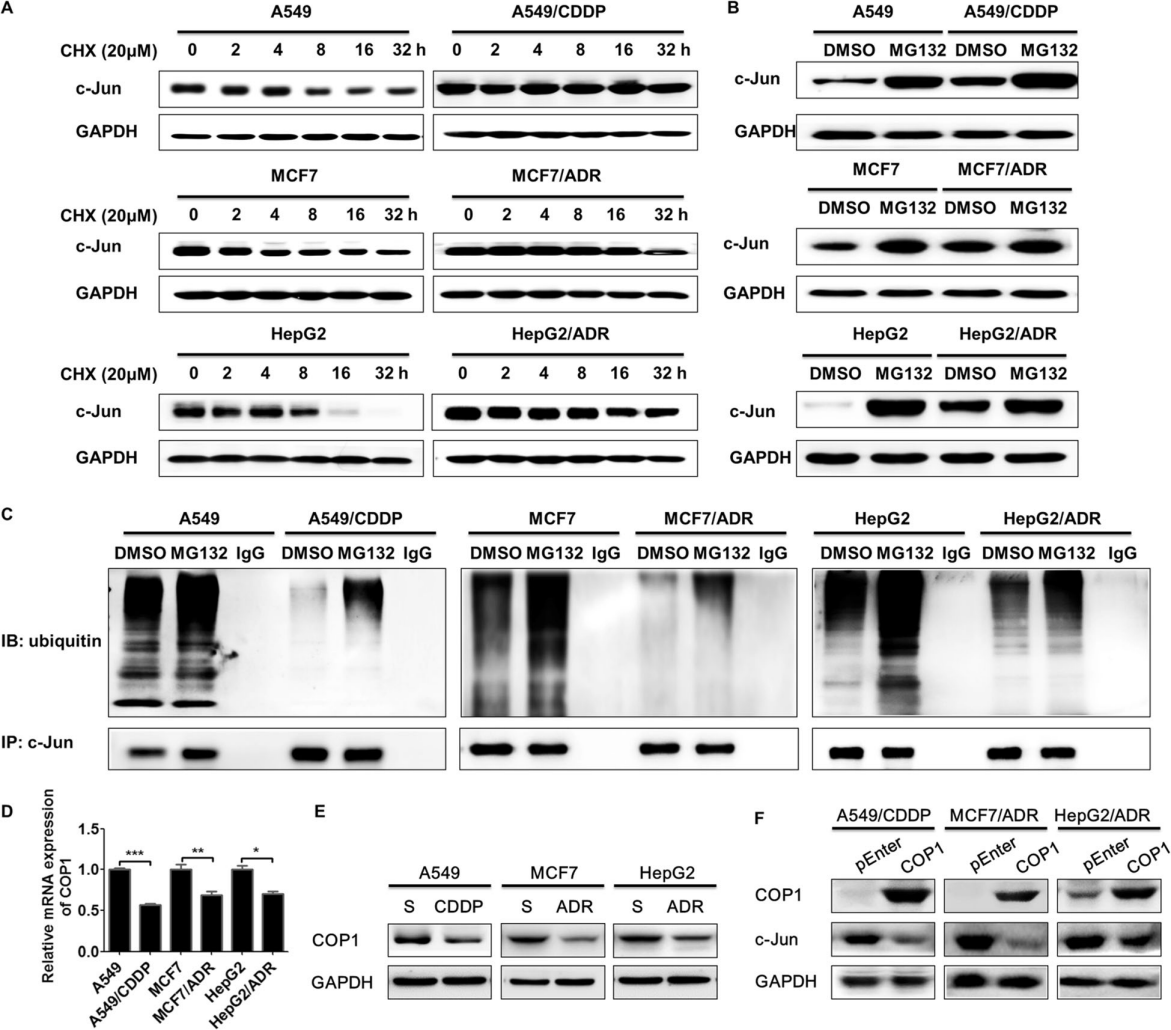
Decreased levels of the E3 ligase COP1 increase c-Jun in drug-resistant A549/CDDP, MCF7/ADR and HepG2/ADR cells. **a** Parental cancer cells and drug-resistant cancer cells were treated with 20 μg/ml cycloheximide (CHX) for the indicated times (0~32 h), and c-Jun expression was detected by western blotting. **b** Parental cancer cells and drug-resistant cancer cells were treated with 10 μM MG132 or solvent DMSO for 8 h, and cellular c-Jun expression was then detected by western blotting. **c** Cells were treated with 10 μM MG132 for 8 h; then, the cells were lysed and immunoprecipitated with c-Jun antibodies or IgG control. Next, the immunoprecipitates were subjected to immunoblot analysis to detect ubiquitin. Parental cancer cells and drug-resistant cancer cells were collected, and COP1 expression was detected by qRT-PCR (**d**) and western blotting (**e**). Drug-resistant cancer cells were transfected with COP1 expression plasmids for 48 h, and COP1 and c-Jun expression was detected by western blotting (**f**). All experiments were performed independently in triplicate. ^*^P ≤ 0.05, ^**^P ≤ 0.01, ^***^P ≤ 0.001

Notably, we observed that total c-Jun protein levels were increased in the drug-resistant cancer cells in our study (Fig. 3a). In addition, c-Jun abundance can be increased by its enhanced protein stability via inhibiting ubiquitin/proteasome-dependent degradation [28]. Thus, we compared the stability and ubiquitination of the c-Jun protein in drug-resistant cancer cells and their parental counterparts to determine the mechanism of c-Jun increase. After blockade of c-Jun protein synthesis by cycloheximide (CHX), c-Jun was degraded more slowly in the drug-resistant cancer cells than in the parental counterparts (Fig. 5a). This suggested a longer half-life period for c-Jun in these drug-resistant cancer cells. Furthermore, it was demonstrated that c-Jun levels were markedly increased after treatment with the proteasome inhibitor MG132 (Fig. 5b), which suggested that c-Jun degradation was ubiquitin/proteasome-dependent. Subsequently, MG132 treatment markedly increased ubiquitin-labeled c-Jun protein levels, and more importantly, c-Jun was less ubiquitinated in the drug-resistant cancer cells (Fig. 5c). These results demonstrated that c-Jun was less ubiquitinated and more stable in drug-resistant A549/CDDP, MCF7/ADR and HepG2/ADR cells.

E3 ligases catalyze the ubiquitination of proteins. Thus, we wondered if the reported E3 ligases, including COP1, cullin 4 (CUL4), F-box and WD repeat domain containing 7 (FBW7), itchy E3 ubiquitin protein ligase (ITCH), mitogen-activated protein kinase kinase kinase 1 (MEKK1), and sensitive to apoptosis gene (SAG), were involved in the ubiquitination of c-Jun [28–34]. Herein, we detected that only COP1 expression was significantly decreased in all drug-resistant cancer cells in our study (Additional file 1: Figure S5, Fig. 5d and e), which suggested the potential role of COP1 in regulating c-Jun abundance via ubiquitination in all drug-resistant cancer cells. Furthermore, COP1 overexpression markedly decreased c-Jun expression in drug-resistant cancer cells (Fig. 5f). The above results collectively demonstrated that decreased COP1 promoted c-Jun protein stability and accumulation in drug-resistant A549/CDDP, MCF7/ADR and HepG2/ADR cells.

### JNK/c-Jun inhibition and HDAC3 overexpression decrease PD-L1 expression in A549/CDDP cells in vivo

**Fig. 6:**
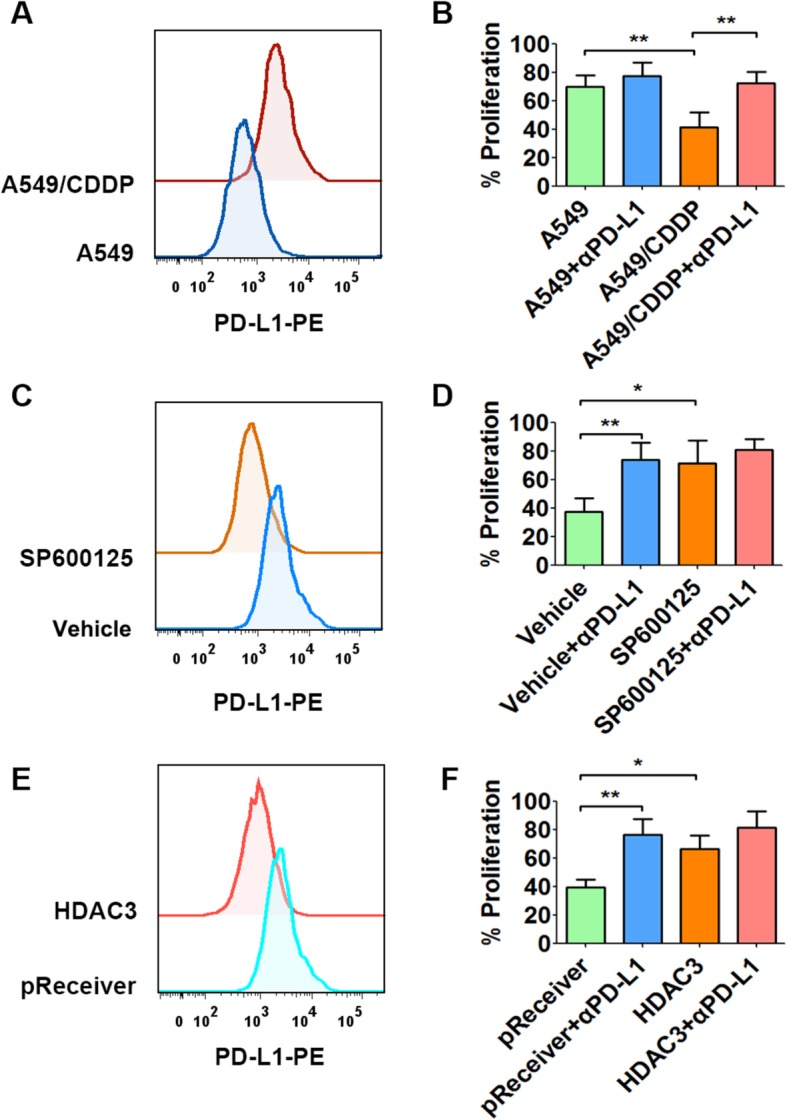
JNK/c-Jun inhibition and HDAC3 overexpression decrease PD-L1 expression in A549/CDDP cells in vivo. A549 and A549/CDDP and A549/CDDP^pReceiver^ and A549/CDDP^HDAC3^ cells were injected into the right flanks of nude mice to form xenograft tumors. When the tumor volumes reached ~ 100 mm^3^, A549/CDDP tumor-bearing mice were treated with PPCES vehicle or SP600125 (15 mg/kg) by intragastrical gavage every 4 days for 2 weeks. The tumors were then collected and digested into single cell suspensions for PD-L1 detection by flow cytometry (**a** and **c**), and T cell proliferation assays were performed (**b** and **d**). When the volumes of A549/CDDP^pReceiver^ and A549/CDDP^HDAC3^ tumors reached ~ 1000 mm^3^, the tumors were collected and digested into single cell suspensions for PD-L1 detection by flow cytometry (**e**), and T cell proliferation assays were performed (**f**). ^*^P ≤ 0.05, ^**^P ≤ 0.01, ^***^P ≤ 0.001

According to the above findings, we next determined whether JNK/c-Jun and HDAC3 were involved in PD-L1 expression in drug-resistant cancer cells (A549/CDDP cells) in vivo using murine xenograft tumor models. It was confirmed that PD-L1 expression was markedly increased in tumor-isolated A549/CDDP cells compared with tumor-isolated A549 cells (Fig. 6a), and this increase could be largely inhibited by oral treatment with SP6000125 (Fig. 6c). Functionally, we detected increased PD-L1 expression in tumor-isolated A549/CDDP cells, which inhibited CD3^+^ T cell proliferation (Fig. 6b), and this effect was largely abolished in A549/CDDP cells isolated from tumors treated with SP6000125 (Fig. 6d). In addition, we found that PD-L1 expression was decreased in stable tumor-isolated A549/CDDP^HDAC3^ cells with high HDAC3 expression compared with tumor-isolated control cells (A549/CDDP^pReceiver^ cells) (Fig. 6e). Furthermore, this PD-L1 decrease promoted CD3^+^ T cell proliferation in vitro (Fig. 6f). The above results collectively demonstrated that JNK/c-Jun and HDAC3 were involved in PD-L1 expression in drug-resistant cancer cells (A549/CDDP cells) in vivo.

### PD-L1 is positively correlated with c-Jun but negatively correlated with HDAC3 expression in cisplatin-resistant NSCLC tissues

**Fig. 7:**
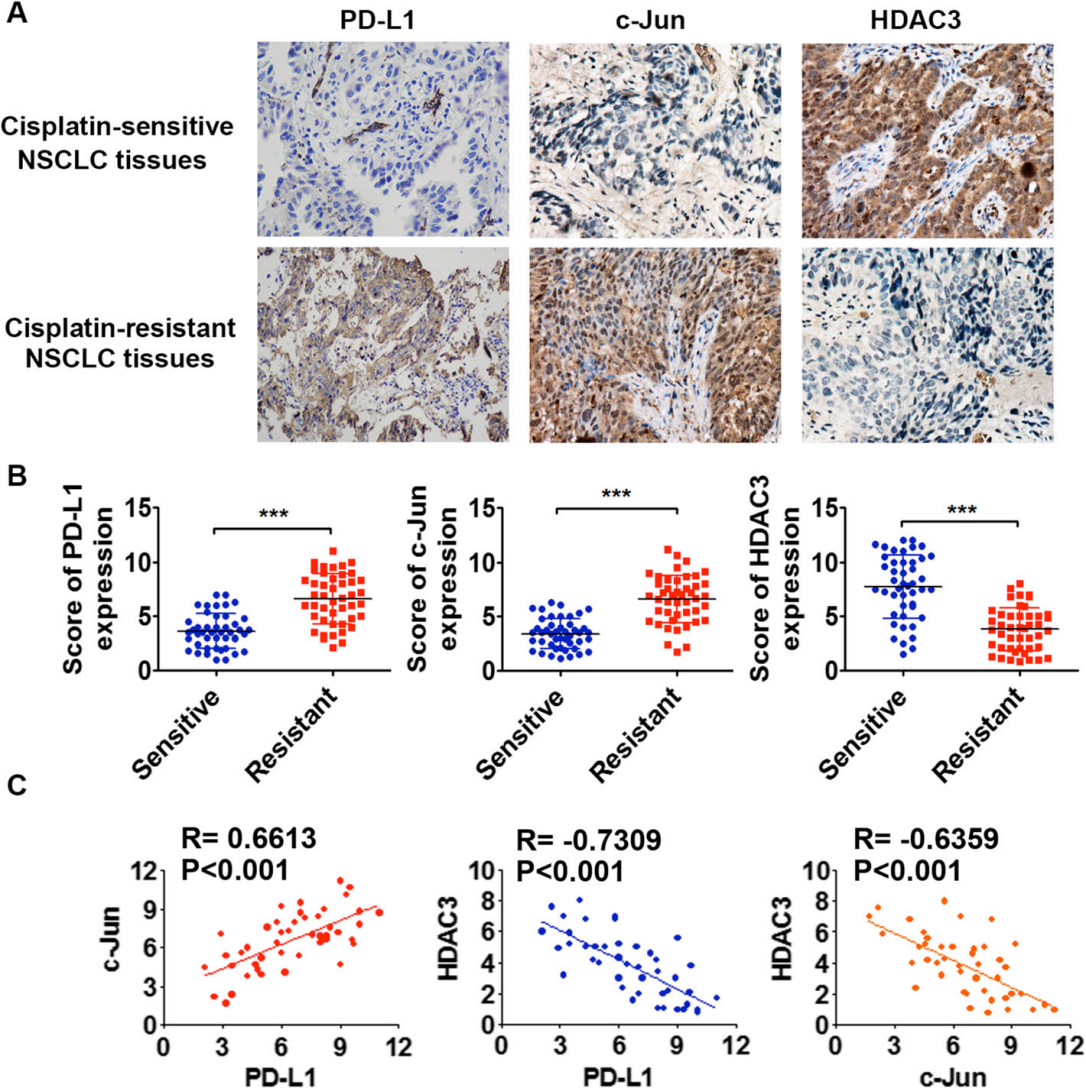
PD-L1 is positively correlated with c-Jun but negatively correlated with HDAC3 expression in cisplatin-resistant NSCLC tissues. The expression of PD-L1, c-Jun and HDAC3 in cisplatin-resistant and cisplatin-sensitive NSCLC tissues was detected by immunohistochemistry. Representative images are shown (**a**) (magnification: 20×). The average scores for PD-L1, c-Jun and HDAC3 expression were analyzed by modified quickscore assessment (**b**). **c** The correlations among PD-L1, c-Jun and HDAC3 expression in cisplatin-resistant NSCLC tumor tissues were analyzed by Pearson’s correlation coefficient. ^*^P ≤ 0.05, ^**^P ≤ 0.01, ^***^P ≤ 0.001

To provide clinical evidence for our above findings, we further analyzed PD-L1, c-Jun and HDAC3 expression in clinical NSCLC tissues sensitive to cisplatin (*n* = 45) and resistant to cisplatin (n = 45). PD-L1 and c-Jun levels were significantly increased, while HDAC3 levels were significantly decreased in the cisplatin-resistant NSCLC tissues compared with the cisplatin-sensitive tumor tissues (Fig. 7a and b). Moreover, we found that PD-L1 was positively correlated with c-Jun expression but negatively correlated with HDAC3 expression, and c-Jun was negatively correlated with HDAC3 expression in cisplatin-resistant NSCLC tissues (Fig. 7c).

## Discussion

As an important immunosuppressor, PD-L1 suppresses the tumor immune response and is associated with poor tumor prognosis [5, 23]. Accumulating studies have demonstrated that acquired resistance to chemotherapeutic agents such as platinum, EGFR-TK inhibitors, and ALK inhibitors is associated with increased PD-L1 in cancer cells [10–12, 35, 36]. In accordance with the above observations, we demonstrated that PD-L1 expression was increased in all three drug-resistant A549/CDDP, MCF7/ADR and HepG2/ADR cells. Clinically, we observed that PD-L1 expression was markedly higher in cisplatin-resistant NSCLC tissues than in cisplatin-sensitive NSCLC tissues. These results confirm the universality of the observation that PD-L1 is increased in drug-resistant cancer cells. Subsequently, we confirmed that increased PD-L1 in the above drug-resistant cancer cells was functionally important to suppress CD3^+^ T cell proliferation, which supports the use of immunotherapy targeting the PD-L1/PD-1 axis to treat drug-resistant cancer cells.

PD-L1 expression in parental cancer cells has been studied extensively [23], while the underlying mechanism of PD-L1 expression in chemoresistant cancer cells remains largely unknown [10, 36]. PD-L1 expression in cancer cells is frequently regulated by a variety of transcription factors, including c-Jun, S6K, STATs and IRF [21]. In this study, we demonstrated that c-Jun plays a crucial role in PD-L1 expression in drug-resistant cancer cells because c-Jun protein knockdown decreased PD-L1 expression in drug-resistant cancer cells, while ectopic c-Jun expression promoted PD-L1 expression in the parental counterparts. Similarly, c-Jun was shown to mediate PD-L1 upregulation in BRAF inhibitor-resistant melanoma cells [15]. Cytokines and growth factors induce JNK phosphorylation and activation, through which c-Jun is subsequently phosphorylated and activated [37, 38]. Next, activated c-Jun is translocated into the nucleus to exert its transcriptional activity [38]. Herein, we demonstrated that JNK/c-Jun signaling activation and nuclear translocation are increased in drug-resistant cancer cells. We subsequently demonstrated that JNK/c-Jun signaling activation markedly increased PD-L1 expression in parental cancer cells, while JNK/c-Jun signaling inhibition dramatically decreased PD-L1 expression in their drug-resistant counterparts. In addition, PD-L1 expression was decreased by JNK/c-Jun signaling inhibition in drug-resistant A549/CDDP cells in vivo, and this could largely reverse the inhibited CD3^+^ T cell proliferation in vitro. These results indicate that JNK/c-Jun signaling is pivotal for PD-L1 expression in drug-resistant cancer cells in vitro and in vivo and could be considered a potential target to improve anticancer immunity in drug-resistant tumors.

Accumulating evidence demonstrates that epigenetic modifications such as DNA methylation and histone acetylation are frequently involved in PD-L1 expression in cancer cells [23, 25]. Methylation of CpG islands over the PD-L1 promoter region negatively regulates PD-L1 transcription [39]. However, our results suggested that promoter DNA methylation was not involved in PD-L1 upregulation in drug-resistant cancer cells in our study. Histone acetylation leads to a more relaxed chromatin structure. Generally, a higher histone acetylation level in the promoter region promotes gene transcription [40]. The HDAC inhibitor LBH589 could rapidly promote PD-L1 expression by increasing histone acetylation of the PD-L1 promoter region in human and mouse melanoma cells [24], which reveals the involvement of histone acetylation modifications in PD-L1 expression. In this study, we demonstrated that histone H3 acetylation of the PD-L1 promoter region was markedly increased in drug-resistant cancer cells. This finding indicates that enhanced histone H3 acetylation of the PD-L1 promoter was involved in PD-L1 expression in drug-resistant cancer cells in our study. Histone H3 acetylation is tightly regulated by HDACs [40]. Among the HDACs, HDAC3 was identified to suppress PD-L1 expression in cancer cells through histone acetylation modifications [27]. In this study, HDAC3 expression was markedly decreased in drug-resistant cancer cells. Furthermore, aberrant HDAC3 expression reversed the increased histone H3 acetylation of the PD-L1 promoter and decreased PD-L1 expression in drug-resistant cancer cells. These results indicate that HDAC3 maintains PD-L1 expression by decreasing histone H3 acetylation of the PD-L1 promoter. In addition, PD-L1 expression in drug-resistant A549/CDDP cells was inhibited by HDAC3 overexpression in vivo, which greatly promoted CD3^+^ T cell proliferation in vitro. These results indicate that HDAC3 inhibits PD-L1 expression in drug-resistant cancer cells in vivo and suggest that HDAC3 activation may be a potential therapeutic approach to reverse the inhibition of T cell-based immunity in drug-resistant tumors.

HDAC3 regulates the acetylation and transcription of c-Jun in cancer cells [41, 42]. However, the role of c-Jun in regulating HDAC3 has rarely been described. In our study, c-Jun knockdown upregulated HDAC3 expression in drug-resistant cancer cells, which indicates the role of negative c-Jun regulation in HDAC3 expression. Furthermore, c-Jun knockdown reversed the increased histone H3 acetylation of the PD-L1 promoter in drug-resistant cancer cells, while c-Jun overexpression increased histone H3 acetylation of the PD-L1 promoter in the parental cells. These results indicate that the c-Jun/HDAC3 axis regulates PD-L1 expression via histone H3 acetylation of the PD-L1 promoter in drug-resistant cancer cells in our study.

c-Jun protein abundance is tightly controlled through a ubiquitin/proteasome-dependent degradation mechanism [43]. Increased protein ubiquitination promotes degradation while decreasing stability and thus shortening the half-life, and vice versa. Herein, we confirmed that c-Jun degradation was ubiquitin/proteasome dependent. Furthermore, the half-life of c-Jun was increased, while c-Jun ubiquitination was markedly decreased, which demonstrated increased c-Jun protein stability in drug-resistant cancer cells. These results indicate that c-Jun protein abundance is increased because of the increased protein stability of c-Jun in the drug-resistant cancer cells. E3 ligases catalyze the linkage of ubiquitin to proteins, and COP1, CUL4, FBW7, ITCH, MEKK1 and SAG are involved in c-Jun ubiquitination. In our study, we identified that COP1 was the main E3 ligase responsible for c-Jun ubiquitination in drug-resistant cancer cells.

To provide clinical evidence for our above findings, we analyzed PD-L1, c-Jun and HDAC3 expression in NSCLC tissues sensitive to cisplatin and resistant to cisplatin. PD-L1 and c-Jun levels were significantly increased, while HDAC3 levels were significantly decreased in cisplatin-resistant NSCLC tissues. Moreover, PD-L1 was positively correlated with c-Jun expression but negatively correlated with HDAC3 expression, and c-Jun was negatively correlated with HDAC3 expression in cisplatin-sensitive NSCLC tissues. These results suggest that our findings from cell studies in vitro and in vivo are clinically significant.

**Fig. 8:**
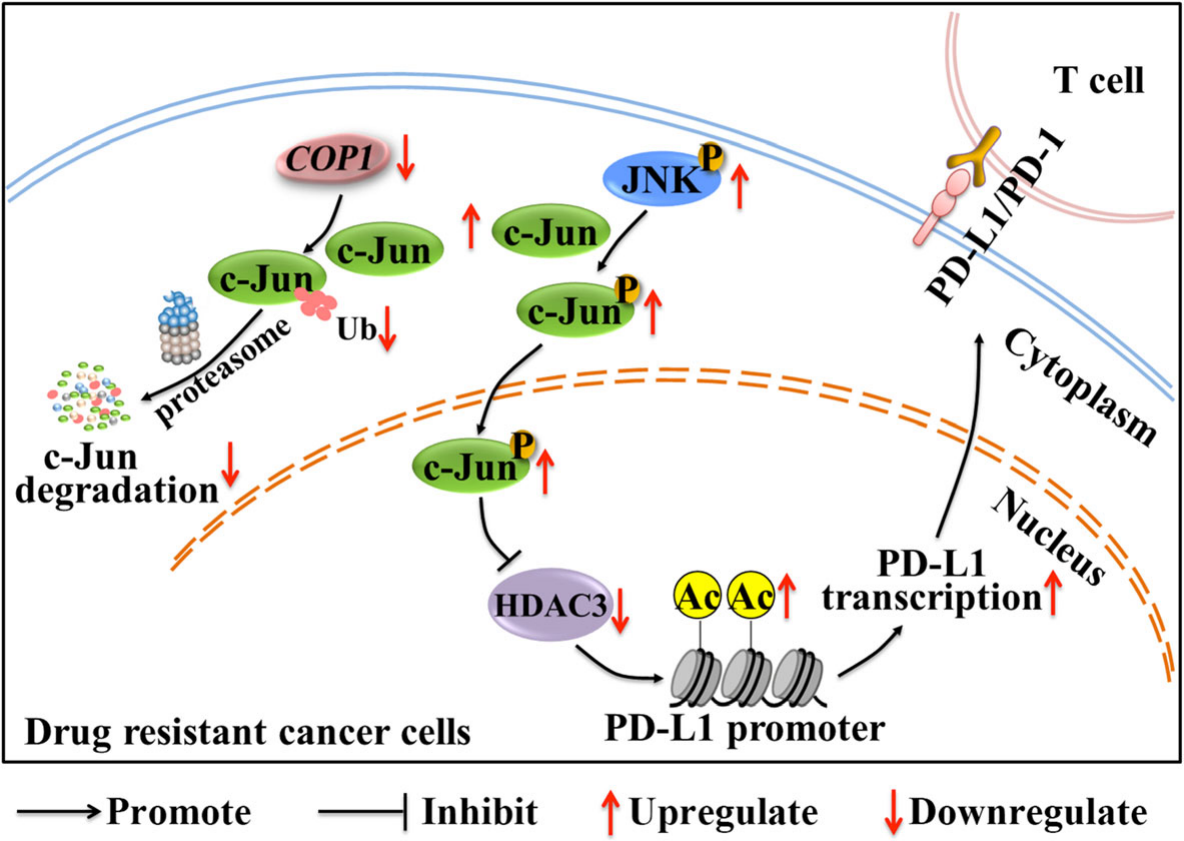
Mechanism proposed to illustrate the PD-L1 increase in drug-resistant A549/CDDP, MCF7/ADR and HepG2/ADR cells. In these drug-resistant cancer cells, decreased levels of the E3 ligase COP1 inhibit c-Jun ubiquitination, which decreases degradation and thereby increases c-Jun accumulation. Then, the increased c-Jun is phosphorylated by JNK and translocated into the nucleus to inhibit HDAC3 expression, which subsequently induces histone H3 acetylation of the PD-L1 promoter. The increased acetylation facilitates PD-L1 transcription and thus increases PD-L1 expression, which suppresses CD3^+^ T cells by interacting with PD-1

In conclusion, we demonstrated that PD-L1 was significantly increased in A549/CDDP, MCF7/ADR and HepG2/ADR cells, and this increase was attributed mainly to enhanced JNK/c-Jun signaling activation. Mechanistically, decreased levels of the E3 ligase COP1 increased c-Jun accumulation, which subsequently inhibited HDAC3 expression and thus increased histone H3 acetylation of the PD-L1 promoter. Furthermore, PD-L1 expression could be inhibited by JNK/c-Jun inhibition or HDAC3 overexpression in vivo, which could largely reverse the inhibited CD3^+^ T cell proliferation in vitro. In clinical NSCLC samples, PD-L1 expression was significantly increased in the cisplatin-resistant group, and PD-L1 expression was positively correlated with c-Jun expression but negatively correlated with HDAC3 expression (Fig. 8). In conclusion, enhanced histone H3 acetylation of the PD-L1 promoter via the COP1/c-Jun/HDAC3 axis was crucial for the PD-L1 increase in drug-resistant cancer cells. Our study reveals a novel regulatory network for the PD-L1 increase in drug-resistant cancer cells and indicates that combined PD-L1-targeting strategies could be used to improve T cell-based immunity in drug-resistant cancers.

## Supplementary information

**Additional file 1:** **Table S1.** Clinical characteristics of the participating patients. **Table S2.** Primer sequences designed for qRT-PCR detection. **Figure S1.** IC_50_ value determination of parental and drug-resistant cancer cells for cisplatin and doxorubicin treatment. **Figure S2.** Identification of the potential regulators involved in PD-L1 expression in drug-resistant A549/CDDP and MCF-7/ADR cells. **Figure S3.** DNA methylation of the PD-L1 promoter is barely changed in drug-resistant A549/CDDP and MCF-7/ADR cells. **Figure S4.** The effects of HDAC inhibitors on PD-L1 expression in drug-sensitive A549, MCF-7 and HepG2 cells. **Figure S5.** Identification of E3 ligases involved in c-Jun expression in drug-resistant A549/CDDP, MCF-7/ADR and HepG2/ADR cells.

## Data Availability

The datasets generated/analysed during the current study are available.

## Abbreviations

ALK: Anaplastic lymphoma kinase
BC: Breast cancer
CFSE: Carboxyfluorescein succinimidyl ester
CHX: Cycloheximide
COP1: Constitutive photomorphogenesis protein 1
CUL4: Cullin 4
DAPI: 4′,6-diamidino-2-phenylindole
EGFR-TK: Epidermal growth factor receptor tyramine kinase
FBW7: F-box and WD repeat domain containing 7
HCC: Hepatocellular carcinoma
HDAC3: Histone deacetylase 3
IRF1: Interferon regulatory factor 1
ITCH: Itchy E3 ubiquitin protein ligase
JNK: c-Jun N-terminal kinases
MEKK1: Mitogen-activated protein kinase kinase kinase 1
NSCLC: Non-small cell lung cancer
PD-L1: Programmed death-ligand-1
S6K: Ribosomal protein S6 kinase
SAG: Sensitive to apoptosis gene
SAHA: Suberoylanilide hydroxamic acid
Stat1: Signal transducers and activators of transcription 1
Stat3: Signal transducers and activators of transcription 3

## References

[1] Bray F, Ferlay J, Soerjomataram I, Siegel RL, Torre LA, Jemal A (2018). Global cancer statistics 2018: GLOBOCAN estimates of incidence and mortality worldwide for 36 cancers in 185 countries. CA Cancer J Clin.

[2] Zahreddine H, Borden KLB (2013). Mechanisms and insights into drug resistance in cancer. Front Pharmacol.

[3] Galluzzi L, Senovilla L, Zitvogel L, Kroemer G (2012). The secret ally: immunostimulation by anticancer drugs. Nat Rev Drug Discov.

[4] Wu T, Dai Y (2017). Tumor microenvironment and therapeutic response. Cancer Lett.

[5] Zou W, Wolchok JD, Chen L (2016). Sci Transl Med.

[6] Wu X, Gu Z, Chen Y, Chen B, Chen W, Weng L (2019). Application of PD-1 blockade in Cancer immunotherapy. Comput Struct Biotechnol J.

[7] Borghaei H, Paz-Ares L, Horn L, Spigel DR, Steins M, Ready NE (2015). Nivolumab versus docetaxel in advanced nonsquamous non-small-cell lung cancer. N Engl J Med.

[8] Planes-Laine G, Rochigneux P, Bertucci F, Chretien AS, Viens P, Sabatier R (2019). PD-1/PD-L1 targeting in breast cancer: the first clinical evidences are emerging. a literature review. Cancers.

[9] El-Khoueiry AB, Sangro B, Yau T, Crocenzi TS, Kudo M, Hsu C (2017). Nivolumab in patients with advanced hepatocellular carcinoma (CheckMate 040): an open-label, non-comparative, phase 1/2 dose escalation and expansion trial. Lancet..

[10] Fujita Y, Yagishita S, Hagiwara K, Yoshioka Y, Kosaka N, Takeshita F (2015). The clinical relevance of the miR-197/CKS1B/STAT3-mediated PD-L1 network in chemoresistant non-small-cell lung cancer. Mol Ther.

[11] Han JJ, Kim DW, Koh J, Keam B, Kim TM, Jeon YK (2016). Change in PD-L1 expression after acquiring resistance to gefitinib in EGFR-mutant non-small-cell lung cancer. Clin Lung Cancer.

[12] Kim SJ, Kim S, Kim DW, Kim M, Keam B, Kim TM (2019). Alterations in PD-L1 expression associated with acquisition of resistance to ALK inhibitors in ALK-rearranged lung cancer. Cancer Res Treat.

[13] Liu J, Liu Y, Meng L, Liu K, Ji B (2017). Targeting the PD-L1/DNMT1 axis in acquired resistance to sorafenib in human hepatocellular carcinoma. Oncol Rep.

[14] Wang Y, Wang L (2017). miR-34a attenuates glioma cells progression and chemoresistance via targeting PD-L1. Biotechnol Lett.

[15] Jiang X, Zhou J, Giobbie-Hurder A, Wargo J, Hodi FS (2013). The activation of MAPK in melanoma cells resistant to BRAF inhibition promotes PD-L1 expression that is reversible by MEK and PI3K inhibition. Clin Cancer Res.

[16] Ishibashi M, Tamura H, Sunakawa M, Kondo-Onodera A, Okuyama N, Hamada Y (2016). Myeloma drug resistance induced by binding of myeloma B7-H1 (PD-L1) to PD-1. Cancer Immunol Res.

[17] Wang HF, Ning F, Liu ZC, Wu L, Li ZQ, Qi YF (2017). Histone deacetylase inhibitors deplete myeloid-derived suppressor cells induced by 4T1 mammary tumors in vivo and in vitro. Cancer Immunol Immunother.

[18] Chen Z, Li K, Yin X, Li H, Li Y, Zhang Q (2019). Lower expression of gelsolin in colon cancer and its diagnostic value in colon cancer patients. J Cancer.

[19] Liu Z, Chen D, Ning F, Du J, Wang H (2018). EGF is highly expressed in hepatocellular carcinoma (HCC) and promotes motility of HCC cells via fibronectin. J Cell Biochem.

[20] Mittendorf EA, Philips AV, Meric-Bernstam F, Qiao N, Wu Y, Harrington S (2014). PD-L1 expression in triple-negative breast cancer. Cancer Immunol Res.

[21] Ritprajak P, Azuma M (2015). Intrinsic and extrinsic control of expression of the immunoregulatory molecule PD-L1 in epithelial cells and squamous cell carcinoma. Oral Oncol.

[22] Dunn C, Wiltshire C, MacLaren A, Gillespie DA (2002). Molecular mechanism and biological functions of c-Jun N-terminal kinase signalling via the c-Jun transcription factor. Cell Signal.

[23] Sun C, Mezzadra R, Schumacher TN (2018). Regulation and function of the PD-L1 checkpoint. Immunity..

[24] Woods DM, Sodre AL, Villagra A, Sarnaik A, Sotomayor EM, Weber J (2015). HDAC inhibition Upregulates PD-1 ligands in melanoma and augments immunotherapy with PD-1 blockade. Cancer Immunol Res.

[25] Darvin P, Sasidharan Nair V, Elkord E (2019). PD-L1 expression in human breast cancer stem cells is epigenetically regulated through posttranslational histone modifications. J Oncol.

[26] Yen CY, Huang HW, Shu CW, Hou MF, Yuan SS, Wang HR (2016). DNA methylation, histone acetylation and methylation of epigenetic modifications as a therapeutic approach for cancers. Cancer Lett.

[27] Deng S, Hu Q, Zhang H, Yang F, Peng C, Huang C (2019). HDAC3 inhibition upregulates PD-L1 expression in B-cell lymphomas and augments the efficacy of anti-PD-L1 therapy. Mol Cancer Ther.

[28] Shao J, Teng Y, Padia R, Hong SG, Noh H, Xie XY (2013). COP1 and GSK3 beta cooperate to promote c-Jun degradation and inhibit breast cancer cell tumorigenesis. Neoplasia..

[29] Migliorini D, Bogaerts S, Defever D, Vyas R, Denecker G, Radaelli E (2011). Cop1 constitutively regulates c-Jun protein stability and functions as a tumor suppressor in mice. J Clin Invest.

[30] Wertz IE, O'Rourke KM, Zhang ZM, Dornan D, Arnott D, Deshaies RJ (2004). Human De-etiolated-1 regulates c-Jun by assembling a CUL4A ubiquitin ligase. Science..

[31] Nateri AS, Riera-Sans L, Da Costa C, Behrens A (2004). The ubiquitin ligase SCFFbw7 antagonizes apoptotic JNK signaling. Science..

[32] Gao M, Labuda T, Xia Y, Gallagher E, Fang D, Liu YC (2004). Jun turnover is controlled through JNK-dependent phosphorylation of the E3 ligase itch. Science..

[33] Xia Y, Wang J, Xu SC, Johnson GL, Hunters T, Lu ZM (2007). MEKK1 mediates the ubiquitination and degradation of c-Jun in response to osmotic stress. Mol Cell Biol.

[34] Gu QY, Bowden GT, Normolle D, Sun Y (2007). SAG/ROC2 E3 ligase regulates skin carcinogenesis by stage-dependent targeting of c-Jun/AP1 and I kappa B-alpha/NF-kappa B. J Cell Biol.

[35] Zhang P, Ma Y, Lv C, Huang M, Li M, Dong B (2016). Upregulation of programmed cell death ligand 1 promotes resistance response in non-small-cell lung cancer patients treated with neo-adjuvant chemotherapy. Cancer Sci.

[36] Lee BS, Park DI, Lee DH, Lee JE, Yeo MK, Park YH (2017). Hippo effector YAP directly regulates the expression of PD-L1 transcripts in EGFR-TKI-resistant lung adenocarcinoma. Biochem Biophys Res Commun.

[37] Grynberg K, Ma FY, Nikolic-Paterson DJ (2017). The JNK signaling pathway in renal fibrosis. Front Physiol.

[38] Zeke A, Misheva M, Remenyi A, Bogoyevitch MA (2016). JNK signaling: regulation and functions based on complex protein-protein partnerships. Microbiol Mol Biol Rev.

[39] Zhang Y, Xiang C, Wang Y, Duan Y, Liu C (2017). PD-L1 promoter methylation mediates the resistance response to anti-PD-1 therapy in NSCLC patients with EGFR-TKI resistance. Oncotarget..

[40] West AC, Johnstone RW (2014). New and emerging HDAC inhibitors for cancer treatment. J Clin Invest.

[41] Zhang L, Shan X, Chen Q, Xu D, Fan X, Yu M (2019). Downregulation of HDAC3 by ginsenoside Rg3 inhibits epithelial-mesenchymal transition of cutaneous squamous cell carcinoma through c-Jun acetylation. J Cell Physiol.

[42] Xia Y, Wang J, Liu TJ, Yung WK, Hunter T, Lu Z (2007). c-Jun downregulation by HDAC3-dependent transcriptional repression promotes osmotic stress-induced cell apoptosis. Mol Cell.

[43] Westermarck J (2010). Regulation of transcription factor function by targeted protein degradation: an overview focusing on p53, c-Myc, and c-Jun. Methods Mol Biol.

