# Supplementary material for: Enhanced histone H3 acetylation of the PD-L1 promoter via the COP1/c-Jun/HDAC3 axis is required for PD-L1 expression in drug-resistant cancer cells
Source: J Exp Clin Cancer Res. 2020 Feb 5;39:29. doi: 10.1186/s13046-020-1536-x (PMC7003365; doi:10.1186/s13046-020-1536-x)
Supplement: Supplementary file 1 — Additional file 1: Table S1. Clinical characteristics of the participating patients. Table S2. Primer sequences designed for qRT-PCR detection. Figure S1. IC50 value determination of parental and drug-resistant cancer cells for cisplatin and doxorubicin treatment. Figure S2. Identification of the potential regulators involved in PD-L1 expression in drug-resistant A549/CDDP and MCF-7/ADR cells. Figure S3. DNA methylation of the PD-L1 promoter is barely changed in drug-resistant A549/CDDP and MCF-7/ADR cells. Figure S4. The effects of HDAC inhibitors on PD-L1 expression in drug-sensitive A549, MCF-7 and HepG2 cells. Figure S5. Identification of E3 ligases involved in c-Jun expression in drug-resistant A549/CDDP, MCF-7/ADR and HepG2/ADR cells. [file 13046_2020_1536_MOESM1_ESM.docx]

**Enhanced histone H3 acetylation of the PD-L1 promoter** **via the COP1/c-Jun/HDAC3 axis is required for PD-L1 expression** **in drug-resistant cancer cells**

Haifang Wang^1, 2, *^, Chen Fu^1, *^, Jun Du^2^, Hongsheng Wang^2^, Rui He^4^, Xiaofeng Yin^1^, Haixia Li^1^, Xin Li^1^, Hongxia Wang^1^, Kui Li^5^, Lei Zheng^1,^ ^‡^, Zongcai Liu^3, ‡^, Yurong Qiu^1, 5, ‡^

**Additional files**

**(Additional file 1: Tables S1-S2 and Figures S1-S5)**

**Table S1.** Clinical characteristics of the participating patients

|  | Cisplatin-sensitive NSCLC patients | Cisplatin-resistant NSCLC patients |
| --- | --- | --- |
| Cases (n) | 45 | 45 |
| Median age (Range) | 63 (43-77) | 67 (48-78) |
| Gender (%) |  |  |
| Male | 35 (78) | 38 (84) |
| Female | 10 (22) | 7 (16) |
| Tumor stage (%) |  |  |
| Stage I | 8 (18) | 6 (13) |
| Stage II | 15 (33) | 13 (29) |
| Stage III | 22 (49) | 26 (58) |
| Stage IV | 0 (0) | 0 (0) |
| Pathological classification (%) |  |  |
| Adenocarcinoma | 28 (62) | 24 (53) |
| Squamous cell carcinoma | 17 (38) | 21 (47) |
| Smoking status (%) |  |  |
| Never | 15 (33) | 10 (22) |
| Former or current | 30 (67) | 35 (78) |

**Table S2.** Primer sequences designed for qRT-PCR detection

|  | Forward primer (5' to 3') | Reverse primer (5' to 3') |
| --- | --- | --- |
| PD-L1 | TGGTGCCGACTACAAGCGAATTAC | TTGGAATTGGTGGTGGTGGTCTTAC |
| S6K | GGATTTCTGGGGAAGAGGTG | TTGGAAGTGGTGCCGATG |
| Stat1 | CACCAGAGCCAATGGAACTT | AGCCCACTATCCGAGACACC |
| Stat3 | ATTCGGAAAGTATTGTCGGC | TGCAGGTCGTTGGTGTCA |
| IRF1 | GATACAAAGCAGGGGAAAAGG | GAAGCATCCGGTACACTCG |
| c-Jun | AGCATGACCCTGAACCTGG | CCGTTGCTGGACTGGATT |
| c-Fos | TCCGAAGGGAAAGGAATAAG | GAGCTGCCAGGATGAACTCT |
| HDAC3 | CCTGGCATTGACCCATAGCC | CTCTTGGTGAAGCCTTGCATA |
| COP1 | TATGCCCCATCTGCTTTGATAT | TTGTCCTCCAAACTCTGATGAA |
| CUL4 | GACCTCACAGTGAACATACTCA | TAGTTTGCCACTGAAGTTTTCG |
| FBW7 | GTCTGAGAACATTAGTGGGACA | ACTTTGAGTGTCCGATCTGTAG |
| ITCH | GCAGCAGTTTAACCAGAGATTC | GTGTGTTGTGGTTGACGAAATA |
| MEKK1 | CTAGAACCTTCAGACCCAATGT | GAGCTACGCCTACTGTGATATT |
| SAG | AACAATCGCTGCCCTCTCT | CTCATTTGCCGATTCTTTGGA |
| GAPDH | GCACCGTCAAGGCTGAGAAC | TGGTGAAGACGCCAGTGGA |


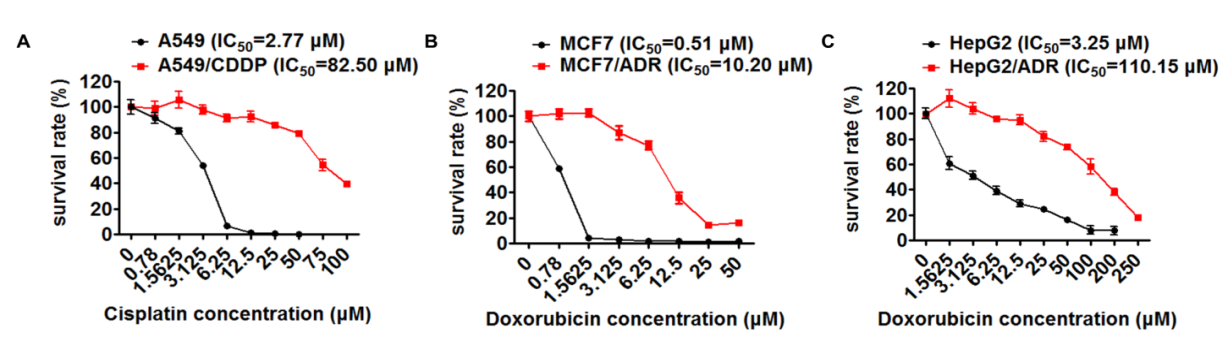


**Figure S1.** IC_50_ value determination of parental and drug-resistant cancer cells for cisplatin and doxorubicin treatment. Drug-sensitive and drug-resistant cancer cells were treated with increasing doses of cisplatin or doxorubicin for 48 h, and cell viability was detected by MTT assay. (A: A549 and A549/CDDP cells, 0 ~ 100 μM cisplatin; B: MCF7 and MCF7/ADR cells, 0 ~ 50 μM doxorubicin; C: HepG2 and HepG2/ADR cells, 0 ~ 250 μM doxorubicin) Next, according to the cell viability detection data, IC_50_ values of the cancer cells for cisplatin and doxorubicin treatment were calculated. All experiments were performed independently in triplicate.


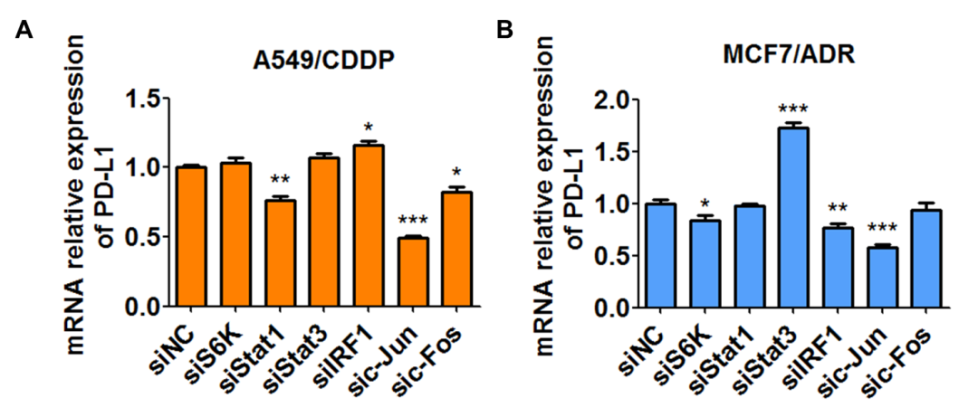


**Figure S2.** Identification of the potential regulators involved in PD-L1 expression in drug-resistant A549/CDDP and MCF-7/ADR cells. Drug-resistant A549/CDDP (A) and MCF7/ADR (B) cells were transfected with siRNAs targeting S6K, Stat1, Stat3, IRF1, c-Jun, and c-Fos or negative control siRNA (siNC) for 24 h, and PD-L1 mRNA expression was detected by qRT-PCR analysis. All experiments were performed independently in triplicate. ^*^P ≤ 0.05, ^**^P ≤ 0.01, ^***^P ≤ 0.001.


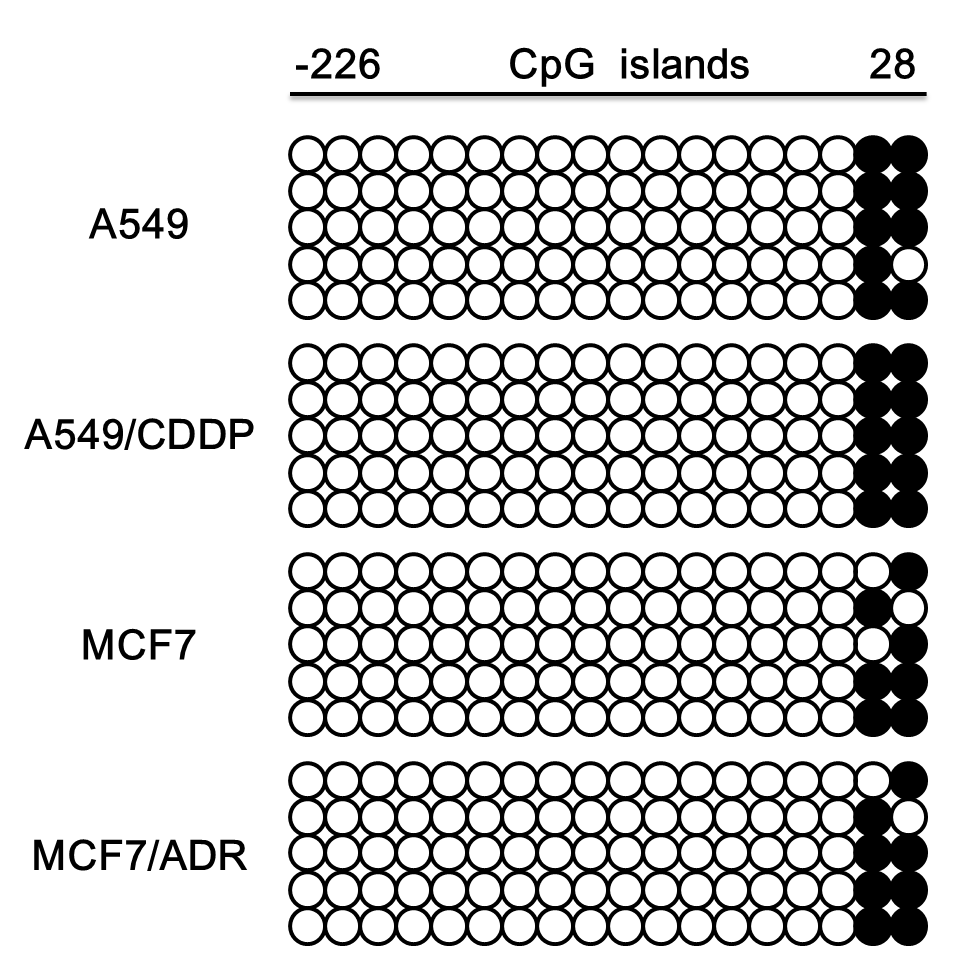


**Figure S3.** DNA methylation of the PD-L1 promoter is barely changed in drug-resistant A549/CDDP and MCF-7/ADR cells. To detect the DNA methylation of the PD-L1 promoter in A549, A549/CDDP, MCF7 and MCF7/ADR cells, genomic DNA was prepared using a TIANamp Genomic DNA kit (Tiangen, Beijing, China), followed by treatment with sodium bisulfite from an Epitect Bisulfite DNA kit (QIAGEN, Germany). Bisulfite-specific primers were designed with Methyl Primer Express software (Applied Biosystems, CA) and used to amplify the promoter region of the PD-L1 gene for both the sense and antisense strands. The sequences of the primers were as follows: forward 5’-GAA AAT TGG ATT GAT ATG TTT TA-3’ and reverse 5’-ACR AAA CCT CRA AAA ACT-3’ (R=A/G). Each dot represents a CpG site. The white dots represent unmethylated CpG dinucleotides, whereas the black dots represent methylated CpG dinucleotides in cytosine residues.


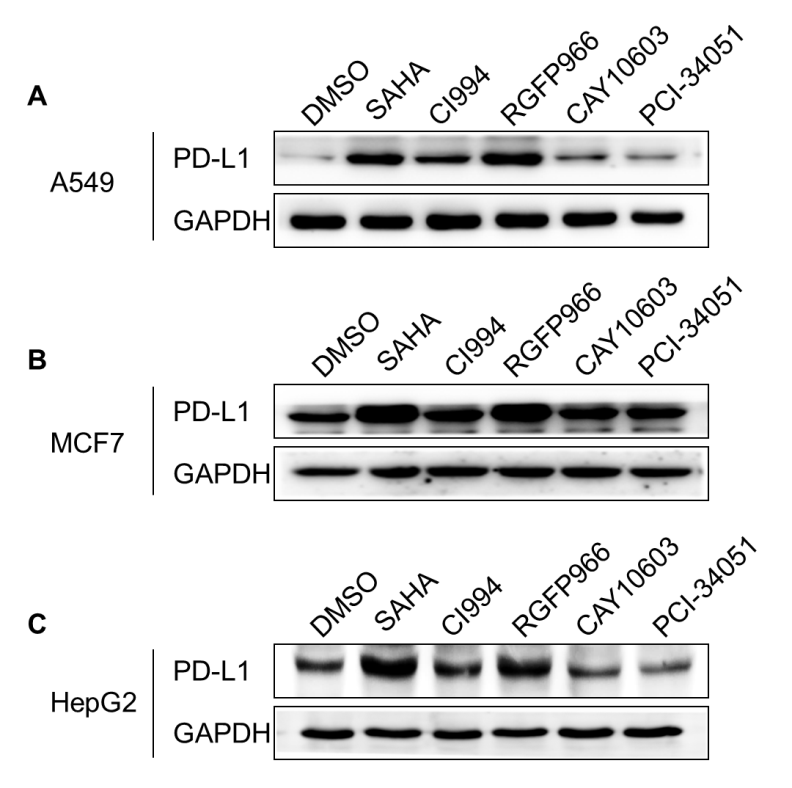


**Figure S4.** The effects of HDAC inhibitors on PD-L1 expression in drug-sensitive A549, MCF-7 and HepG2 cells. A549 (A), MCF7 (B), and HepG2 (C) cells were treated with a pan-HDAC inhibitor (SAHA, 2 μM), HDAC1-specific inhibitor (CI994, 2 μM), HDAC3-specific inhibitor (RGFP966, 2 μM), HDAC6-specific inhibitor (CAY10603, 2 μM), HDAC8-specific inhibitor (PCI-34051, 2 μM), or solvent DMSO for 48 h, and PD-L1 protein expression was detected by western blotting.


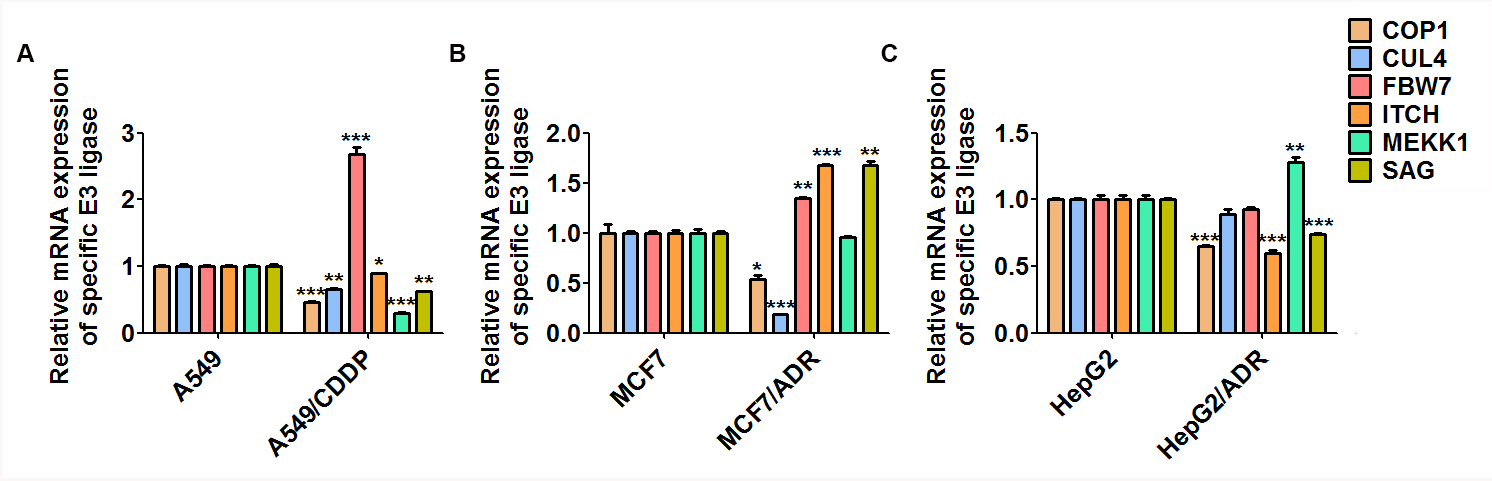


**Figure S5.** Identification of E3 ligases involved in c-Jun expression in drug-resistant A549/CDDP, MCF-7/ADR and HepG2/ADR cells. Drug-sensitive and resistant cancer cells were collected, and E3 ligases, including COP1, CUL4, FBW7, ITCH, MEKK1, and SAG, were detected by qRT-PCR (A: A549 and A549/CDDP cells; B: MCF7 and MCF7/ADR cells; C: HepG2 and HepG2/ADR cells). All experiments were performed independently in triplicate. ^*^P ≤ 0.05, ^**^P ≤ 0.01, ^***^P ≤ 0.001.
